# The Bidirectional Relationship Between Rheumatoid Arthritis and Periodontitis: Current Evidence and Clinical Implications

**DOI:** 10.7759/cureus.116330

**Published:** 2026-09-15

**Authors:** Naif A Alwithanani

**Affiliations:** 1 Department of Oral and Maxillofacial Surgery and Diagnostic Science, Taif University, Taif, SAU

**Keywords:** bidirectional relationship, inflammation, oral-systemic health, periodontal therapy, periodontitis, rheumatoid arthritis

## Abstract

Rheumatoid arthritis (RA) and periodontitis are chronic inflammatory diseases that share several pathogenic, immunological, and environmental characteristics. Increasing evidence suggests that these conditions are linked through a potentially bidirectional relationship in which chronic periodontal inflammation may contribute to the initiation and progression of RA, whereas RA may predispose individuals to periodontal tissue destruction through systemic inflammation, immune dysregulation, and impaired oral hygiene. This narrative review summarizes the current understanding of the epidemiological association between RA and periodontitis and discusses the shared inflammatory pathways, including cytokine dysregulation, osteoclast activation, and altered host immune responses, that underlie both diseases. Particular emphasis is placed on the reciprocal influence of periodontitis on RA activity and the impact of RA on periodontal disease. Available evidence indicates that periodontal infection may amplify the systemic inflammatory burden and autoimmune responses, whereas chronic inflammatory changes associated with RA can accelerate periodontal tissue breakdown and increase the risk of disease progression. Emerging clinical evidence also suggests that periodontal therapy may serve as a valuable adjunct to conventional RA management by reducing the systemic inflammatory burden and improving disease activity, although definitive causal relationships have not yet been established. Periodontal therapy may have beneficial effects on systemic inflammatory markers and selected measures of RA activity, although the clinical significance and consistency of these effects remain uncertain. Recognition of this bidirectional association highlights the importance of interdisciplinary collaboration between dental professionals and rheumatologists to facilitate early diagnosis, comprehensive patient assessment, and development of integrated treatment strategies. Future longitudinal and interventional studies are warranted to clarify the causal mechanisms linking these conditions and determine whether improved periodontal health can contribute to better long-term outcomes in patients with RA.

## Introduction and background

Rheumatoid arthritis (RA) is a chronic, systemic autoimmune inflammatory disorder characterized by persistent synovial inflammation, progressive cartilage destruction, and irreversible bone erosion, ultimately leading to joint deformities and functional disability [1]. Recent epidemiological estimates indicate a substantial and potentially increasing burden of RA in India, with women disproportionately affected, and projections indicate that the disease burden will continue to increase in the coming years, emphasizing the need for early diagnosis and comprehensive management [2]. Although the disease primarily affects synovial joints, accumulating evidence indicates that RA is a multisystem disorder with significant extra-articular manifestations, including adverse effects on oral health [3].

Periodontitis is a chronic, biofilm-associated inflammatory disease characterized by dysbiosis of the subgingival microbial community and a dysregulated host immune-inflammatory response, resulting in progressive destruction of the periodontal attachment apparatus and alveolar bone [4]. It is among the most prevalent chronic diseases worldwide and shares several epidemiological and pathogenic characteristics with RA, including chronic inflammation, immune dysregulation, genetic susceptibility, and progressive connective tissue and bone destruction [5]. Over the past two decades, growing interest has focused on the biological relationship between these seemingly distinct diseases, leading to the recognition of a potential bidirectional association between them.

Although smoking, age, obesity, diabetes mellitus, and socioeconomic and behavioral factors may partly explain the observed association, evidence of overlapping immunological and inflammatory pathways provides additional biological plausibility for an independent relationship [6]. Both conditions are characterized by exaggerated host immune responses involving pro-inflammatory cytokines, including tumor necrosis factor-α (TNF-α), interleukin (IL)-1β, IL-6, and IL-17, as well as activation of the receptor activator of nuclear factor kappa-B (RANK) ligand (RANKL) pathway, which promotes osteoclastogenesis and bone resorption [7]. Furthermore, the periodontal pathogen *Porphyromonas gingivalis (P. gingivalis)* has attracted considerable attention because of its unique ability to express bacterial peptidylarginine deiminase (PPAD), an enzyme capable of citrullinating host proteins. This process has been proposed to contribute to the generation of anti-citrullinated protein antibodies (ACPAs), which are among the most specific serological markers of RA and are implicated in its pathogenesis [8]. These findings support the hypothesis that chronic periodontal infection may contribute to the initiation or progression of ACPA-positive RA in genetically susceptible individuals.

The mucosal origins hypothesis of RA has further stimulated interest in the oral cavity as a potential site of immune dysregulation, particularly because periodontal inflammation can promote protein citrullination, microbial translocation, and systemic inflammatory signaling [9]. Multiple observational studies and systematic reviews have reported a higher prevalence and/or severity of periodontitis among individuals with RA, although the magnitude of the association varies across studies [6,7,10]. Emerging interventional evidence suggests that non-surgical periodontal therapy may reduce systemic inflammatory markers and may produce modest improvements in selected measures of RA disease activity; however, the clinical significance and consistency of these effects remain uncertain [11]. This narrative review summarizes the current evidence regarding the bidirectional relationship between RA and periodontitis, with particular emphasis on shared pathogenic mechanisms, epidemiological associations, the impact of periodontal therapy on RA outcomes, and the clinical implications for collaborative patient management by dental and medical professionals.

## Review

A comprehensive literature search was conducted across PubMed/MEDLINE, Scopus, and Web of Science for articles published up to May 2026, using combinations of the keywords "rheumatoid arthritis", "periodontitis", "periodontal disease", "citrullination", "*Porphyromonas gingivalis*", "PPAD", "ACPA", "RANKL", and "bidirectional relationship", combined using Boolean operators (AND/OR). Reference lists of relevant articles were hand-searched to identify additional studies. Eligible sources included original research, systematic reviews, and meta-analyses published in English addressing epidemiological, mechanistic, or interventional aspects of the RA-periodontitis association. Editorials, conference abstracts, and non-peer-reviewed sources were excluded. Given the narrative design, no formal quality-appraisal tool or quantitative synthesis was applied.

For this narrative review, relevant literature was identified through a focused search of published studies addressing the epidemiological association, shared pathogenic mechanisms, bidirectional effects, and clinical implications of RA and periodontitis. Priority was given to systematic reviews and meta-analyses, randomized controlled trials, cohort and case-control studies, and relevant experimental and mechanistic investigations, supplemented by established narrative reviews where appropriate. Studies were considered based on their relevance to the review objectives, methodological contribution, and ability to inform the major thematic areas. The available evidence was categorized according to the principal topic addressed, including epidemiological associations, shared inflammatory and immunological mechanisms, the influence of periodontitis on RA, the influence of RA on periodontal health, and therapeutic and clinical implications. 

Epidemiological association between RA and periodontitis

Among the various etiological and contributing factors implicated in periodontal disease, the association between periodontitis and RA has been extensively investigated. Multiple observational studies and systematic reviews have reported a higher prevalence and/or greater severity of periodontitis among individuals with RA, although the magnitude of this association varies across populations and study designs [6,7,10,12]. Similarly, patients with periodontitis appear to have an increased risk of developing RA, suggesting a reciprocal relationship that extends beyond shared environmental and lifestyle risk factors [12]. Although the exact temporal sequence remains incompletely understood, available epidemiological evidence supports a biologically plausible association between the two chronic inflammatory conditions.

Periodontitis affects approximately 40-50% of adults globally, whereas RA has a prevalence of approximately 0.5-1% [13,14]. The interpretation of epidemiological associations is complicated by substantial overlap in risk factors, including smoking, age, obesity, diabetes, socioeconomic status, healthcare access, and oral-health behaviors. Although several studies have reported associations after adjustment for selected confounders, residual confounding cannot be excluded [6,7]. Smoking is particularly relevant because it increases the risk and severity of periodontitis and is also associated with enhanced mucosal protein citrullination and increased risk of ACPA-positive RA, particularly in genetically susceptible individuals [15]. The previous literature has reported a significant association between RA and periodontitis, even after adjusting for smoking as a confounder, suggesting that common pathogenic mechanisms independently contribute to disease coexistence [16].

Although correlations between periodontal parameters and RA disease activity have been reported, these observations do not establish the directionality of the relationship because systemic inflammation, shared risk factors, treatment status, and reverse causation may contribute to the observed association [6,7,17]. Conversely, individuals with moderate to severe periodontitis have been reported to exhibit an elevated risk of RA, particularly among those with seropositive disease characterized by the presence of ACPAs or rheumatoid factor [18]. Although variations in study design, diagnostic criteria, and population characteristics contribute to heterogeneity across studies, the overall direction of evidence consistently supports a positive association between these conditions.

Disease severity also appears to be correlated between these two disorders. Patients with active RA frequently exhibit more severe periodontal destruction, whereas severe periodontitis has been associated with higher RA disease activity scores, elevated serum C-reactive protein (CRP) concentrations, and increased circulating levels of pro-inflammatory cytokines [17]. These observations suggest that persistent periodontal inflammation may contribute to the systemic inflammatory burden, whereas chronic systemic inflammation associated with RA may accelerate periodontal tissue breakdown [7].

However, epidemiological studies cannot establish causality because most available evidence originates from cross-sectional and case-control investigations. Differences in periodontal case definitions, RA classification criteria, medication use, and patient demographics further limit direct comparisons between the studies [17]. Nevertheless, the consistency of epidemiological observations across diverse populations, together with the accumulating mechanistic and interventional evidence, strengthens the concept of a clinically relevant bidirectional relationship between RA and periodontitis. Future well-designed longitudinal cohort studies are required to clarify temporal associations and determine whether effective prevention or treatment of one condition can reduce the incidence or progression of the other condition.

Shared pathogenic mechanisms

The biological association between RA and periodontitis is supported by several shared pathogenic mechanisms involving chronic inflammation, immune dysregulation, and osteoimmunological pathways [18]. Although these diseases affect different anatomical sites, they exhibit striking similarities in the cellular and molecular processes responsible for connective tissue destruction and bone resorption. These common mechanisms provide a plausible explanation for their frequent coexistence and bidirectional influences [19].

Both RA and periodontitis are characterized by an exaggerated host immune response driven by the persistent activation of innate and adaptive immune cells. Macrophages, neutrophils, dendritic cells, and activated T and B lymphocytes produce excessive amounts of pro-inflammatory cytokines, including TNF-α, IL-1β, IL-6, and IL-17 [20]. These inflammatory mediators promote matrix degradation and disturb the RANKL-RANK-osteoprotegerin (OPG) axis, thereby favoring osteoclast differentiation and bone resorption [21].

Although the PPAD-citrullination hypothesis provides a compelling mechanistic link, experimental and observational findings have not established that *P. gingivalis* infection is necessary or sufficient for the development of RA in humans [22]. In genetically susceptible individuals, citrullinated proteins may trigger the production of ACPAs, which are highly specific to RA and play a central role in disease initiation and progression [23]. This has led to the hypothesis that chronic periodontal infection may contribute to the breakdown of immune tolerance and promote autoimmune responses before the clinical onset of RA [18].

Genetic susceptibility further strengthens the biological association between these two diseases. Human leukocyte antigen (HLA)-DRB1 shared epitope alleles are strongly associated with ACPA-positive RA and may interact with environmental factors, such as smoking and periodontal infection, to enhance protein citrullination and autoantibody formation [24]. Both diseases are polygenic disorders in which variations in immune-related genes may influence the magnitude of the inflammatory responses. A case-control study by Schulz et al. [25] identified polymorphisms in interferon-γ (IFN-γ), TNF-α, and IL-4 receptor-α (IL4Rα) as potential genetic markers associated with the comorbidity of RA and severe periodontitis, suggesting that shared genetic determinants may predispose susceptible individuals to both diseases.

Collectively, these findings suggest that RA and periodontitis are interconnected through common inflammatory and immune pathways, rather than representing isolated diseases [26]. Understanding these shared mechanisms not only explains their epidemiological association but also provides a biological rationale for integrated management strategies aimed at reducing systemic inflammation and improving outcomes in both conditions.

How periodontitis influences RA

Growing evidence suggests that periodontitis may contribute to the initiation and progression of RA through the systemic dissemination of inflammatory mediators and periodontal pathogens [7,26]. Chronic periodontal inflammation may result in recurrent or transient episodes of bacteremia, particularly in association with periodontal inflammation and routine oral activities, and may contribute to elevated circulating levels of pro-inflammatory cytokines, including TNF-α, IL-1β, and IL-6, thereby potentially amplifying systemic inflammation and exacerbating synovial disease activity [12,19]. This chronic inflammatory burden is considered one of the principal mechanisms linking periodontal disease to increased RA severity.

Among periodontal microorganisms, *P. gingivalis* has attracted particular attention because of its unique expression of PPAD, an enzyme capable of citrullinating host proteins [27]. Citrullinated proteins can trigger the production of ACPAs, which are highly specific to RA and are implicated in disease initiation and progression [22]. Experimental evidence further demonstrated that animals infected with a PPAD-deficient strain of *P. gingivalis* exhibited significantly reduced periodontal inflammation, lower serum ACPA levels, and less severe arthritis than those infected with the wild-type strain, supporting the contribution of bacterial PPAD to both periodontal disease and RA [28]. However, these findings were obtained from experimental animal models of arthritis and should not be considered directly equivalent to human RA. Although they provide mechanistic support for a potential link between periodontal infection and autoimmune responses, their direct applicability to the initiation or progression of human RA remains uncertain. These findings therefore provide a plausible biological mechanism linking periodontal infection to autoimmune responses potentially involved in RA.

Clinical observations further support this association. Patients with untreated periodontitis often exhibit higher RA disease activity scores, elevated CRP concentrations, and increased erythrocyte sedimentation rate (ESR) compared with those with healthy periodontal tissues [29]. Furthermore, several interventional studies have demonstrated that non-surgical periodontal therapy can modestly reduce systemic inflammatory markers and improve the Disease Activity Score in 28 joints (DAS28), suggesting that effective periodontal treatment may complement conventional RA management [30,31]. Although periodontal therapy should not be considered a substitute for disease-modifying antirheumatic drugs (DMARDs), current evidence suggests that periodontal therapy may provide an adjunctive benefit in patients with RA, particularly with respect to systemic inflammatory markers and selected measures of disease activity; however, the magnitude, durability, and clinical relevance of this effect remain uncertain.

A recent systematic review and meta-analysis of 10 randomized controlled trials involving 430 patients demonstrated that non-surgical periodontal therapy was associated with significant short-term reductions in DAS28-CRP, while improvements in DAS28-ESR showed a similar trend but with greater uncertainty due to study heterogeneity [32]. Although the available evidence remains limited by relatively small sample sizes, methodological heterogeneity, and short follow-up periods, these findings suggest that periodontal therapy may serve as a valuable adjunct to conventional pharmacological management by reducing the systemic inflammatory burden rather than replacing DMARDs. Overall, current evidence suggests that chronic periodontal inflammation is not merely a local oral condition but may actively influence the onset, severity, and clinical course of RA through immunological and inflammatory mechanisms.

How RA influences periodontitis

The relationship between RA and periodontitis is reciprocal, with RA predisposing patients to poor periodontal health. Chronic systemic inflammation associated with RA alters immune function and bone metabolism, creating an environment that favors periodontal tissue destruction [6,7]. In a case-control study, Pischon et al. [33] reported substantially higher odds of periodontitis among individuals with RA after adjustment for selected confounders; however, the magnitude of this association should be interpreted in the context of the study design and population. These findings indicate that the association between RA and periodontitis cannot be explained solely by inadequate plaque control, suggesting the contribution of shared inflammatory and immunological mechanisms.

The persistent production of pro-inflammatory cytokines, including TNF-α, IL-1β, and IL-6, contributes to enhanced osteoclast differentiation through the activation of the RANKL pathway. The resulting imbalance between bone resorption and bone formation accelerates alveolar bone loss and increases susceptibility to periodontal breakdown [26,29,34]. Additionally, immune dysregulation in RA may impair the host's ability to maintain periodontal homeostasis, leading to an exaggerated inflammatory response to subgingival biofilms.

Functional limitations associated with RA also contribute to the deterioration of oral health. Pain, joint stiffness, and deformities affecting the hands and wrists may reduce manual dexterity, making routine oral hygiene practices, such as tooth brushing and interdental cleaning, more difficult. Inadequate plaque control subsequently promotes bacterial accumulation and the progression of periodontal disease [35]. Furthermore, xerostomia resulting from associated conditions, such as secondary Sjögren's syndrome, or from certain medications, may compromise oral health by reducing salivary protective functions. Reduced salivary flow may increase the risk of dental caries, mucosal discomfort, and oral dryness, and may make oral hygiene and plaque control more difficult. Although these changes may potentially contribute to periodontal inflammation, the direct contribution of xerostomia to periodontal tissue destruction remains less clearly established [36,37].

Pharmacological management of RA may also influence periodontal status. Effective treatment with conventional DMARDs and biologic agents can suppress systemic inflammation and may indirectly improve periodontal health by reducing the activity of inflammatory cytokines [38]. However, despite advances in RA therapy, patients remain at an increased risk of periodontal disease, emphasizing the need for regular periodontal assessment and preventive care as part of comprehensive disease management.

Overall, the available evidence indicates that RA contributes to periodontal disease through a combination of systemic inflammation, immune dysregulation, altered bone metabolism, and functional impairment. These findings reinforce the concept that successful management of RA should include routine periodontal evaluation and close collaboration between rheumatologists and dental professionals to minimize the impact of both chronic inflammatory diseases [38,39].

Limitations

Although the available evidence supports an association between RA and periodontitis, several limitations should be considered when interpreting the findings of this narrative review. First, the literature considered was restricted to English-language publications, which may introduce publication and language bias and result in the omission of relevant evidence published in other languages. Second, as a narrative review, the selection and interpretation of studies inevitably involve author judgment, introducing the possibility of selection bias. Third, shared risk factors, including smoking, aging, obesity, diabetes mellitus, and socioeconomic factors, remain important potential confounders and may partly contribute to the observed association between RA and periodontitis. In addition, substantial heterogeneity exists across studies with respect to periodontal and RA case definitions, RA disease activity and serological status, medication use, periodontal treatment, smoking status, and follow-up duration, limiting direct comparisons and synthesis of findings. Reverse causation is also an important consideration, particularly in cross-sectional studies, in which the temporal sequence between RA and periodontitis cannot be established. Finally, evidence derived from experimental animal and mechanistic studies should not be considered equivalent to evidence from longitudinal human studies, as findings from experimental models provide biological plausibility but have limited direct applicability to human RA initiation or progression. These limitations highlight the need for well-designed longitudinal and interventional studies using standardized diagnostic criteria and outcome measures to clarify the temporal and causal relationships between RA and periodontitis.

Clinical implications and future perspectives

Routine periodontal examination should be incorporated into the comprehensive management of patients with RA, followed by appropriate periodontal diagnosis and individualized risk assessment. Patients diagnosed with periodontitis should receive non-surgical periodontal therapy when indicated, together with appropriate oral hygiene education and risk-factor management. Because both RA and periodontitis are chronic inflammatory conditions, periodontal care should not be limited to initial screening or treatment. Supportive periodontal therapy and regular maintenance should be continued to monitor periodontal stability, reinforce preventive measures, and identify recurrent or progressive disease at an early stage. Likewise, effective control of systemic inflammation through appropriate RA management may contribute to improved periodontal health [38]. This integrated approach may help reduce the long-term inflammatory burden and support continued collaboration between rheumatologists and dental professionals.

Dentists may be among the first healthcare professionals to identify patients who may warrant rheumatological evaluation, particularly when severe or rapidly progressing periodontitis is accompanied by persistent inflammatory joint symptoms, morning stiffness, symmetric small-joint involvement, unexplained functional limitation, or a relevant medical history suggestive of systemic inflammatory disease [30]. Periodontal findings alone should not be considered diagnostic of RA; rather, the combination of periodontal and relevant systemic clinical features may support timely referral for appropriate medical evaluation.

Routine periodontal screening, patient education regarding oral hygiene, and timely referral to periodontal specialists may help reduce the inflammatory burden associated with chronic periodontal infection [40]. Similarly, effective control of systemic inflammation through appropriate RA management may contribute to improved periodontal health [38]. This collaborative approach aligns with the growing emphasis on integrated care for chronic inflammatory diseases, recognizing oral health as an important component of overall systemic health.

Although current evidence supports an association between RA and periodontitis, several questions remain unanswered. The temporal relationship between these two diseases, the influence of specific periodontal pathogens on RA initiation, and the long-term effects of periodontal therapy on RA progression require further investigation. Future longitudinal cohort studies and well-designed randomized clinical trials with standardized diagnostic criteria and outcome measures are needed to clarify the causality and identify patient populations that may derive the greatest benefit from integrated management strategies.

Advances in molecular biology and microbiome research may further improve our understanding of the mechanisms linking periodontal dysbiosis with systemic autoimmunity. The identification of shared biomarkers and therapeutic targets could facilitate early diagnosis, personalized treatment approaches, and improved prevention strategies for individuals at risk of both diseases. Therefore, continued collaboration between periodontology and rheumatology is essential to translate emerging evidence into improved patient care.

## Conclusions

RA and periodontitis are chronic inflammatory diseases that are increasingly recognized to share a complex bidirectional relationship mediated by common genetic, immunological, and inflammatory pathways. Chronic periodontal inflammation may contribute to the initiation and progression of RA through the systemic dissemination of inflammatory mediators and citrullinated antigens. RA may, in turn, increase susceptibility to periodontal destruction through immune dysregulation, enhanced osteoclast activity, and impaired oral hygiene resulting from functional disability. Although current evidence supports this reciprocal association, definitive causal relationships remain to be established through well-designed longitudinal and interventional studies with large sample sizes. Nevertheless, the available data underscore the importance of interdisciplinary collaboration between rheumatologists and dental professionals. Early periodontal screening, timely intervention, and integrated patient management may help reduce the overall inflammatory burden, improve oral and systemic health outcomes, and enhance the quality of life of individuals affected by chronic inflammatory disorders.
